# Accidental breakage of needle during subclavian vein catheterization: an adversity uncalled for!

**DOI:** 10.5935/0103-507X.20170056

**Published:** 2017

**Authors:** Sadik Mohammed, Swati Chhabra, Pradeep Kumar Bhatia, Manoj Kamal, Pooja Bihani

**Affiliations:** Department of Anaesthesiology, All India Institute of Medical Science - AIIMS, Jodhpur, Rajasthan, India.; Department of Anaesthesiology, All India Institute of Medical Science - AIIMS, Jodhpur, Rajasthan, India.; Department of Anaesthesiology, All India Institute of Medical Science - AIIMS, Jodhpur, Rajasthan, India.; Department of Anaesthesiology, All India Institute of Medical Science - AIIMS, Jodhpur, Rajasthan, India.; Department of Anaesthesiology, All India Institute of Medical Science - AIIMS, Jodhpur, Rajasthan, India.

To the editor,

Central venous catheter (CVC) placement is an essential component of modern-day critical care. Its use was first documented by Werner Forssman in 1929, and Seldinger perfected the technique in 1953.^(1)^ Although CVC insertion is a routine practice, it is not devoid of complications. We report a rare event in which the introducer needle shaft was fractured from its hub during CVC insertion in the right subclavian vein.

A 60-year-old male patient was admitted to the intensive care unit with septic shock. In the course of management, central venous catheterization of right subclavian vein was planned. After informed and written consent was obtained, necessary preparations were made. Initial ultrasound screening demonstrated a collapsible subclavian vein situated 4cm deep in the skin. The triple lumen CVC set (Romsons Scientific & Surgical Industries Pvt. Ltd., Agra, India. M/s, Romodex U. K. Ltd.) was assessed and flushed with heparinized saline. A Y-type introducer needle (18G; 70mm) was inserted under ultrasound guidance using an in-plane approach. Successful venous puncture required insertion of almost the full length of the needle, and the J-tipped guide wire was then inserted through the other port of the 'Y'. When a sufficient length of the guide wire was inserted, needle removal was attempted. At this time, the needle shaft was fractured and separated from hub syringe assembly (Figure 1A). The hub syringe assembly was removed, and the visible tip of the fractured needle was grasped using hemostatic forceps. Maintaining constant outward pressure on the forceps and inward pressure on the guide wire, the fractured needle was removed carefully. The position of the guide wire was again confirmed with ultrasound, and the path was dilated with a dilator. CVC was railroaded over the guide wire, and the guide wire was removed. Close inspection revealed that the needle shaft was intact, and its breakage from the hub was the cause of the event (Figure 1B).

**Figure 1:**
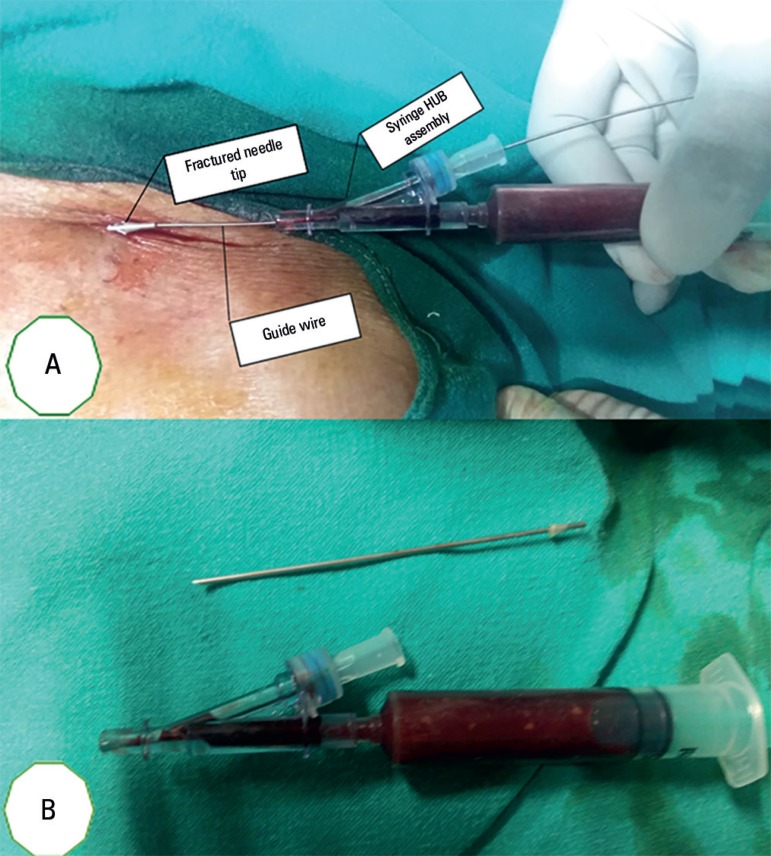
(A) Tip of the introducer needle with the guide wire in place. (B) Close inspection of the introducer needle and hub syringe assembly.

Complications associated with CVC insertion fluctuate according to their definition and correlation with the multiple factors. Greater than 15% of patients experience catheter-related complications.^(2)^ Mechanical complications occur in 5 - 19% of patients, infectious complications in 5 - 26% and thromboembolic complications in 2 - 26%.^(3)^ Designing institutional standardized methods of CVC insertion is a logical process to promote prevention and decrease the incidence of complications. At our center, we routinely perform visual inspection of individual components of the CVC set and flush the catheter with saline prior to insertion. Additionally, all CVCs are inserted under ultrasound guidance, which allows any undue angulation of the needle to be avoided especially during subclavian vein catheterization. In spite of all of these pre-use measures, we faced this adversity due to faulty equipment. In our case, a portion of the needle could be grasped; otherwise, it would have been a great ordeal if the needle was lost in the subcutaneous tissue. Botolin et al. reported similar event during placement of the CVC in the left subclavian vein. However, in this case, the needle was lost in the subcutaneous tissue, and its retrieval required multi-disciplinary intervention, including radiology, critical care, vascular surgery, and thoracic surgery.^(4)^

Our case and the only other published report by Botolin et al.^(4)^ clearly imply that routine assessments of the equipment should be performed; however, this safety measure will not preclude all mishaps. Measures should be taken to prevent such unwanted events by using ultrasound guidance as a routine practice and to avoid extreme angulations while attempting to puncture the subclavian vein during catheterization.

 Sadik Mohammed *Department of Anaesthesiology, All India Institute of Medical Science -
AIIMS, Jodhpur, Rajasthan, India.* Swati Chhabra *Department of Anaesthesiology, All India Institute of Medical Science -
AIIMS, Jodhpur, Rajasthan, India.* Pradeep Kumar Bhatia *Department of Anaesthesiology, All India Institute of Medical Science -
AIIMS, Jodhpur, Rajasthan, India.* Manoj Kamal *Department of Anaesthesiology, All India Institute of Medical Science -
AIIMS, Jodhpur, Rajasthan, India.* Pooja Bihani *Department of Anaesthesiology, All India Institute of Medical Science -
AIIMS, Jodhpur, Rajasthan, India.*

## References

[1] Seldinger SI (1953). Catheter replacement of the needle in percutaneous arteriography;
a new technique. Acta Radiol.

[2] Merrer J, De Jonghe B, Golliot F, Lefrant JY, Raffy B, Barre E, Rigaud JP, Casciani D, Misset B, Bosquet C, Outin H, Brun-Buisson C, Nitenberg G, French Catheter Study Group in Intensive Care (2001). Complications of femoral and subclavian venous catheterization in
critically ill patients: a randomized controlled trial. JAMA.

[3] Alemohammad M (2013). Central venous catheter insertion problem solving using
intravenous catheter: technical communication. Tehran Univ Med J.

[4] Botolin D, Mooser A, Stillions D, Mortman K, Sarin S, Babrowicz J (2015). Needle loss in subclavian vein during central venous catheter
placement: case report of a rare complication. Patient Saf Surg.

